# Pathogenesis of Alzheimer’s Disease

**DOI:** 10.3390/ijms24010107

**Published:** 2022-12-21

**Authors:** Agueda A. Rostagno

**Affiliations:** Department of Pathology, Grossman School of Medicine, New York University, New York, NY 10016, USA; agueda.rostagno@nyulangone.org

Alzheimer’s disease (AD) is the most common type of dementia, accounting for 60% to 80% of all cases. It is estimated that this debilitating neurodegenerative disorder currently affects 50 million patients worldwide and indirectly impacts the lives of tens of millions who deal with many years of cognitive decline in their loved ones [1]. A small percentage of all AD cases are linked to dominant genetic mutations in three genes codifying for *APP* (amyloid precursor protein), *PSEN1* (presenilin 1), and *PSEN2* (presenilin 2) and are typically associated with early-onset forms of the disease in which clinical symptoms appear before the age of 65 [2,3]. The majority of the patients exhibit late-onset Alzheimer’s disease (LOAD), which appears later in life and is sporadic in nature. Although in these cases the disease is not hereditary and shows no single genetic cause, current evidence supports the existence of a number of genetic risk factors, among which the presence of the E4 allele in the *ApoE* (apolipoprotein E) gene—occurring in about 16% of the population—is the most significant [4]. Lifestyle behaviors such as poor diet and reduced physical activity, as well as environmental and metabolic risk factors including diabetes, cerebrovascular disease, head injury, and stress, are typically linked with an increased risk for the disease [3,5,6].

The deposition of amyloid-β (Aβ) in the brain parenchyma and the cerebral vasculature, together with the presence of intraneuronal neurofibrillary tangles and the gradual loss of synapses, are central neuropathological hallmarks of AD, although, to this day, it remains unclear what primarily triggers and drives the progression of the disease [3,7]. In spite of the more than 100 years that have passed since the discovery of the disease, the complex molecular mechanisms leading to the pathophysiology of AD have still not been fully elucidated. Among the different multifactorial pathways affected by the disease, vascular abnormalities, mitochondrial detrimental changes, oxidative stress, reduced brain glucose utilization, and neuroinflammation are currently being considered as important players in the initiation and/or progression of the disease. 

This Special Issue of *International Journal of Molecular Sciences* (*IJMS*) contains four original research articles and six reviews authored by a panel of experts that highlight the recent advances in the current knowledge of the molecular mechanisms contributing to the pathogenesis of multifactorial AD. The research articles cover a wide range of topics, from the influence exerted by Western diets and apoE genotypes, the analysis of Aβ-mediated changes in synaptic dysfunction, and the upregulation of the COVID-19 receptor in the brain of AD patients, to the development of new animal models. Mattar et al. explored the contribution of Western-style dietary patterns to the genetic risk imposed by the ApoE4 allele [8]. Western diets, characterized by a prolonged intake of saturated fats and sugars, strongly increase the risk of diabetes and cardiovascular disease, which affects AD susceptibility [9]. In Mattar’s current study, homozygous male and female ApoE4 knock-in mice subjected to dietary formulations with elevated levels of saturated fats and sucrose showed metabolic alterations and behavioral impairments. The abnormalities were aggravated in male animals, which also exhibited liver dysfunction and high levels of inflammatory cytokines in the brain. Notably, female ApoE4 mice exposed to the same Western-style diet showed impairments in spatial learning and memory, but without liver dysfunction or increased brain inflammatory markers. The findings underscore the multiple direct and indirect pathways through which genetic and diet-related factors interact, highlighting striking sex differences in markers of chronic neuroinflammation, and providing a note of caution in the need to consider gender as a crucial variable for the interpretation of research data.

Back M. et al. focused on the neocortex, a brain region that is affected very early in AD, and—in line with patients’ findings—in the 5xFAD AD mouse that shows spine and neuron loss in the neocortex earlier than in the hippocampus [10]. The authors investigated whether the specific expression and/or regulation of N-methyl-D-aspartate receptor (NMDAR) subunits contributed to the early neuronal Aβ-toxicity in the neocortex by assessing in vitro NMDAR-mediated currents in neocortical neurons with virus-mediated Aβ-overexpression, as well as in vivo in neocortical neurons of 5xFAD mice. The contribution of NMDAR subunits to Aβ-toxicity for neocortical neurons was evaluated using conditional GluN1, GluN2B, and 5xFADxGluN1/GluN2B knockout mice. Overall, the research provides novel insights into the mechanisms of Aβ-mediated changes in the synaptic function of neocortical neurons and their dependence on NMDA receptors, which are known to play important roles in learning and memory and are critical for spatial memory [11].

The studies by Ding Q. et al. linked AD with the coronavirus disease 2019 (COVID-19) pandemic. Infection with the severe acute respiratory syndrome coronavirus 2 (SARS-CoV-2), which uses angiotensin-converting enzyme 2 (ACE2) as a receptor to enter the host cells, is frequently associated with neurological manifestations [12,13]. The present study showed the upregulated protein expression of ACE2 in the brain of AD patients and demonstrated a moderate positive correlation between these increased levels and the presence of oxidative stress, as indicated by the elevated presence of carbonylated proteins and the enhanced thiol oxidation state of peroxiredoxin 6 [14]. Overall, the study highlights the relationships between AD and the receptor for SARS-CoV-2, underscoring the importance of carefully monitoring patients with both Alzheimer’s disease and COVID-19 to identify higher viral loads and long-term adverse neurological consequences in the brain.

Zampar and Wirths characterized a novel AD mouse model that was generated to evaluate the relationship between the two most prominent neuropathological hallmarks of the disease, the extracellular Aβ deposition and the intracellular accumulation of hyperphosphorylated tau in neurofibrillary tangles, in the absence of confounding human APP overexpression. The authors focused on the N-terminally truncated Aβ4–42 peptides, one of the most abundant Aβ proteoforms in the amyloid deposits, with the aim of understanding whether the presence of these peptides affected the onset or extent of tau pathology. To achieve these goals, the authors crossed the homozygous Tg4–42 mouse model of AD, exclusively expressing Aβ4–42 peptides, with the PS19 (P301S) tau transgenic model [15]. Although no significant difference in the tau pathology was observed in the resulting line in comparison with the PS19, a behavioral assessment showed that the double-transgenic line presented a partial worsening of motor performance and spatial memory deficits in the aged group. Although an increased loss of distal CA1 pyramidal neurons was detected in young mice, no significant alterations in hippocampal tau phosphorylation were observed in immunohistochemical analyses.

The review articles cover several of the underlying mechanisms participating in the Alzheimer’s pathogenesis, including selective autophagy and mitochondrial dysfunction, the contribution exerted by the activation of the NLRP3 inflammasome, and the role played by insulin and insulin resistance. Topics addressed by these review articles also summarized imaging techniques employed in the early diagnosis and longitudinal monitoring of AD, as well as the use of metal-containing compounds for positron emission tomography (PET), magnetic resonance imaging (MRI), and single-photon emission computed tomography (SPECT). Sedzikowska et al. reviewed the role played by insulin in the CNS, both in healthy individuals and those affected with pathologies associated with insulin resistance and AD. The emphasis of the article was centered in two signal transduction pathways: the PBK/AKT pathway, responsible for the metabolic effects of insulin, and the MAPK pathway, which influences cell growth, cell survival and gene expression [16]. Overall, the authors highlighted the role exerted by brain insulin resistance in the development and progress of AD, including the increase in oxidative stress, production of Aβ42, and Tau protein phosphorylation, which, in turn, impair mitochondrial function, cognition, and memory. Litwiniuk et al. expanded on the issue of insulin resistance in the Alzheimer’s pathogenesis by incorporating chronic inflammation, increased oxidative stress, and impaired energy metabolism as features shared by AD, obesity, and type-2 diabetes. Particular emphasis was given to the activation of the inflammasome complex, specifically NLRP3, as well as to the release of proinflammatory cytokines IL-1β and IL-18 [17]. Based on the influence of neuroinflammatory processes exerted by NLRP3, the authors postulated that this signaling pathway may underlie the association between adiposity, insulin resistance, and cognitive impairment.

Two of the review articles focused on diagnostic imaging methodologies. van Oostveen et al. summarized the utility, benefits, and limitations of multiple imaging techniques and associated biomarkers in the early diagnosis and longitudinal monitoring of AD. The manuscript discussed the value of using radiotracers to assess hippocampal volume, hypometabolism in temporoparietal brain regions, and Aβ and Tau accumulation, emphasizing their limitations regarding specificity, reliability, and sensitivity [18]. Since current tools require relatively high levels of protein accumulation or neurodegeneration to be detectable, the authors emphasized the need to identify novel biomarkers that reflect AD pathogenesis at the earliest stages as essential for the proper development of disease-modifying therapies. Krasnovskaya et al. expanded on the use of imaging techniques, particularly in new metal-containing radiopharmaceuticals for AD visualization aimed to avoid the current radiotracers based on ^11^C and ^18^F that are synchrotron-dependent and short-lived. The authors reviewed the requirements for the development of coordination compounds capable of crossing the blood–brain barrier, and summarized metal-containing drugs for PET, MRI, and SPECT that are suitable for imaging in AD [19]. 

Finally, two of the review articles focused on two different crucial mechanistic pathways and their contribution to AD pathogenesis. Sharma et al. delved into the growing evidence implicating mitochondrial dysfunction as a common driver of cognitive impairment in AD. They presented an overview of the importance of mitochondria for the maintenance of neuronal function and their role in the pathological mechanisms involved in many of the hallmark features of AD, including the formation of Aβ aggregates, neurofibrillary tangles, synaptic transmission plasticity, oxidative stress, and neuroinflammation. Dysregulation in mitochondrial function, motility, fission, and fusion leads to neuronal malfunction and degeneration associated with excess free radical production and reduced intracellular calcium buffering. They also provide a brief summary of possible treatments targeting mitochondrial dysfunction as potential therapeutic approaches for AD [20]. Guan et al. provided mechanistic insights into the process of autophagy, a mechanism that ensures the timely degradation of damaged cellular components in eukaryotic cells. In recent years, different subtypes of autophagy have been identified; in their article, the authors extensively discussed the underlying mechanisms governing these various subtypes, with the long-term goal of understanding how to control the process to treat AD [21]. Since the evidence overwhelmingly suggests that selective autophagy is an active contributing mechanism in AD pathology, the regulation of this autophagy subtype could constitute an effective strategy for modulating AD pathogenesis.

Overall, this Special Issue underscores the multifactorial nature of AD and the complexity of the pathways affected in the disease, highlighting the need to simultaneously target multiple risk factors and disease mechanisms at an early stage and emphasizing the urgency of developing new diagnostic strategies for a more accurate and early diagnosis.
